# Integration of mathematical model predictions into routine workflows to support clinical decision making in haematology

**DOI:** 10.1186/s12911-020-1039-x

**Published:** 2020-02-10

**Authors:** Katja Hoffmann, Katja Cazemier, Christoph Baldow, Silvio Schuster, Yuri Kheifetz, Sibylle Schirm, Matthias Horn, Thomas Ernst, Constanze Volgmann, Christian Thiede, Andreas Hochhaus, Martin Bornhäuser, Meinolf Suttorp, Markus Scholz, Ingmar Glauche, Markus Loeffler, Ingo Roeder

**Affiliations:** 1 Institute for Medical Informatics and Biometry, Faculty of Medicine Carl Gustav Carus, Technische Universität Dresden, Dresden, Germany; 2 Institute for Medical Informatics, Statistics and Epidemiology, Faculty of Medicine, University of Leipzig, Leipzig, Germany; 3 Abteilung Hämatologie/Onkologie, Klinik für Innere Medizin II, Universitätsklinikum Jena, Jena, Germany; 4 Department of Internal Medicine, Medical Clinic I, University Hospital Carl Gustav Carus Dresden, Dresden, Germany; 5 Pediatric Hematology and Oncology, Department of Pediatrics, University Hospital Carl Gustav Carus Dresden, Dresden, Germany; 6 National Center for Tumor Diseases (NCT), Partner Site Dresden, Dresden, Germany

**Keywords:** Clinical decision-making, Support system, Data management, Individual therapy planning, Routine workflow, Haematology, Model-based treatment optimization, Mathematical modelling, Computer simulation

## Abstract

**Background:**

Individualization and patient-specific optimization of treatment is a major goal of modern health care. One way to achieve this goal is the application of high-resolution diagnostics together with the application of targeted therapies. However, the rising number of different treatment modalities also induces new challenges: Whereas randomized clinical trials focus on proving average treatment effects in specific groups of patients, direct conclusions at the individual patient level are problematic. Thus, the identification of the best patient-specific treatment options remains an open question. Systems medicine, specifically mechanistic mathematical models, can substantially support individual treatment optimization. In addition to providing a better general understanding of disease mechanisms and treatment effects, these models allow for an identification of patient-specific parameterizations and, therefore, provide individualized predictions for the effect of different treatment modalities.

**Results:**

In the following we describe a software framework that facilitates the integration of mathematical models and computer simulations into routine clinical processes to support decision-making. This is achieved by combining standard data management and data exploration tools, with the generation and visualization of mathematical model predictions for treatment options at an individual patient level.

**Conclusions:**

By integrating model results in an audit trail compatible manner into established clinical workflows, our framework has the potential to foster the use of systems-medical approaches in clinical practice. We illustrate the framework application by two use cases from the field of haematological oncology.

## Background

The availability of highly effective cytotoxic agents, tumour-specific drugs, and other targeted therapy options are the mainstay of treatment for many cancer types. Typically, combinations of treatment modalities are administered to achieve an optimal response. Furthermore, supportive measures complement the anti-tumour treatment to mitigate toxic side effects, thereby improving overall treatment success. As the number of therapeutic options for many cancers rises, treatment optimization becomes more challenging. Whereas randomized clinical trials can provide objective evidence of benefit for a group of patients, they typically do not allow conclusions at the individual patient level. Although risk group stratification can be useful, the identification of the best patient-specific treatment options, such as type and dosage of drugs, remains an open question.

Computational tools and mechanistic mathematical modelling can substantially support individual treatment optimization by patient-specific model predictions. This especially applies for disease and treatment dynamics that result from a complex interplay of individual disease pathologies (e.g. tumour aggressiveness, chemo-sensitivity, pharmacokinetics and –dynamics of anticancer drugs, risk factors), which are difficult to predict empirically. Hence, we sought to establish a number of disease and treatment models for haematological malignancies, such as high-grade Non-Hodgkin’s lymphomas (NHL) ([1–4]) and chronic myeloid leukaemia (CML) ([5–8]). In addition to providing a better general understanding of the disease mechanisms and treatment effects, these models identify patient-specific parameterizations, which are essential to provide individually tailored predictions.

For routine clinical decision making, these models have to be usable by a broad clinical community. Furthermore, the model results have to be integrated with many other clinical parameters. In current clinical practice, physicians typically extract diagnostic and staging information from a multitude of data sources. Basic clinical information, including diagnostic parameters, or details about potential therapies (e.g., drug type, dosing, response and side effects) are frequently stored in different and potentially heterogeneous systems (e.g. medical information systems, device-specific data bases, laboratory systems, *in-house* semi-integrated and department-specific solutions, and often still in paper-based medical records). Such decentralized data storage makes information retrieval and clinical appraisal a complicated, cumbersome process.

Physicians need to integrate all this information with results from previous examination, new diagnostic results, and their personal experience. A structured presentation together with suitable visualization of data can potentially help this process. Current database interfaces usually present medical data in text/table format, whereas graphical visualization is uncommon, yet. However, it could improve assessment of disease status and how it changes over time. Moreover, decisions about future developments, e.g. whether to alter treatment schedules, are difficult because they are often influenced by many disease- and therapy-related and individual factors. Mathematical models may potentially help with this.

Here, we demonstrate how mathematical models can be integrated into routine clinical workflows. This comprises processing of input data, simulation of alternative treatment scenarios, user-friendly presentation of clinical data and model results, as well as suggestions for individualized treatment schedules. Besides the technical description of the framework architecture, i.e. the linking of different software applications and data flows, we demonstrate how simulated results can be integrated in database front-ends to allow easy access in a software prototype (see demo server at https://hopt.imb.medizin.tu-dresden.de and Additional file 3).


**Additional file 3** Demo server video tutorial.


## Implementation

### Requirement analysis

The starting point of our prototype development was the analysis of requirements in everyday clinical practice. In close collaboration with the University Hospitals Dresden and Jena, the established processes of collecting data from NHL and CML patients were analysed and documented in use case diagrams. We identified a number of existing weaknesses in the routine workflow (such as distributed clinical systems, multiple data collection, heterogeneous / redundant datasets) and formulated the needs to improve or even eliminate these in the future. Based hereon, we defined a list of necessary software features (Additional file 1). Furthermore, we analyzed and described the technical requirements of the computational models to be implemented regarding administration, required access to patient data, execution of simulations, deployment of patient specific simulation results and presentation to clinicians in an easily and unambiguously interpretable fashion. All resulting insights have been summarized in *entity relationship diagrams* (Additional file 2), which were the basis for the database development.

### Software architecture

**Fig. 1:**
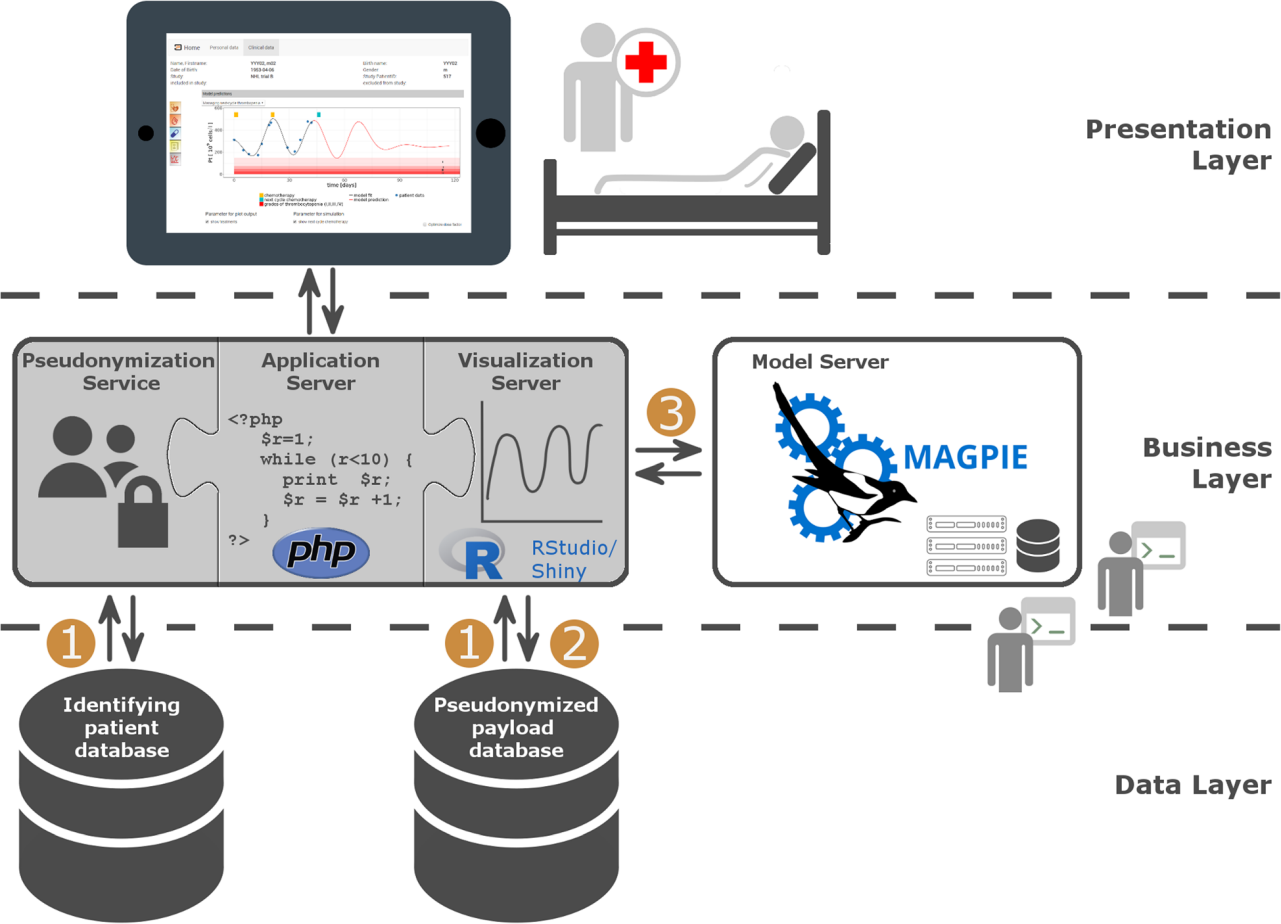
Software Architecture. The *data layer* comprises two relational databases to store patient identifying data and pseudonymized payload data separately. The *business layer* adds an application server with a pseudonymization service, a visualization server, as well as a server supporting model simulations (MAGPIE). In particular, the application server provides the access to patient identifying data and pseudonymized payload data (1). The visualization server is strictly separated from the identifying patient database and exclusively retrieves medical data from the pseudonymized payload database for data description and model prediction (2 and 3). The *presentation layer* provides the frontend with a web-based graphical user interface for onsite access by physicians. The php- and R-logo are taken from the websites http://php.net/download-logos.php and https://www.r-project.org/logo/. Both images are under the terms of the Creative Commons and Attribution-Share Alike 4.0 International (CC-BY-SA 4.0)

Based on the requirement analysis, a multi-layer architecture was developed (see Fig. 1). In the *data layer*, we applied two relational databases (Database Management System: Microsoft SQL Server 2008 R2 [9]) for storing (a) patient identifying data and (b) pseudonymized medical data (payload data) separately. To provide transparency and reproducibility, both databases contain stored procedures for all operations that are used by software tools of the *business layer*. The *business layer* comprises different components: (i) an application server with pseudonymization service implemented in the server-side scripting languages PHP 7 [10] and JavaScript running on an Apache HTTP Server, (ii) a visualization server using RStudio’s Shiny package [11], and (iii) the MAGPIE model server [12] for model management and execution based on the web-application framework Ruby on Rails [13] running on the webserver Nginx [14]. For a detailed description of the MAGPIE framework and implementation we refer the reader to Baldow et al. 2017 [12]. On top of the data and business layer, a *presentation layer* has been implemented in form of a browser accessible web-based graphical user interface (GUI) for an easy access and onsite use by physicians.

### Frontend and backend design

The frontend was designed for an optimal display on desktop and tablet. Depending on access rights, the user interface menu provides access to patient identifying data, pseudonymized or de-pseudonymized medical data, core data, access rules, and user settings.

*Patient identifying data* contain sensitive person-related data that are stored in an identifying patient database (c.f. section: Data protection). *Core data* (see also Results) are master data that comprise basic information about relevant objects (e.g. drugs, units, diagnostic parameters, hospitals, etc.). *Medical data* contain pseudonymized (i.e. non-identifying) patient-specific information like treatment details, diagnostic parameters, and diagnoses. *Core data* and *medical data* are stored in a *pseudonymized payload*^1^ *database* (see Fig. 1).

We designed the tables of the payload database with the goal that every type of medical data can be stored in a common, harmonized form. To substantially reduce manual effort for structurally new data, we use “long tables”, i.e. saving the type of data along with the data themselves in a separate column, avoiding manual adding of additional columns to the database. In addition to presenting medical data in table form, we developed several interactive *Shiny* applications to visualize data (e.g. time courses) and embedded them in the graphical user interface (GUI) via the html element *iframe*.

The following three principle backend workflows were established (c.f. Fig. 1).

1. *Display and editing of patient identifying data and medical data*: The application server with the pseudonymization service connects to the identifying patient database and to the payload database via open database connectivity (ODBC) using stored procedures, and retrieves data according to pre-defined user permissions.
2. *Visualization of medical data:* Whenever medical data is visualized, the *Shiny* server connects to the payload database via ODBC and retrieves the necessary medical data via stored procedures. To keep security standards as high as possible, the *Shiny* server is strictly separated from the database with patient-identifying information. The reversal of the pseudonymization is realized by the pseudonymization service of the webserver.
3. *Provision of model predictions*: To generate model simulations and to present corresponding predictions, we use *Shiny* applications together with the MAGPIE framework [12], serving as a backend computation platform. MAGPIE provides online and remote access to deployed computational models and supports their parametrization and execution. Technically, every simulation request within the *Shiny* application results in an internal action of the *Shiny* server to check whether the particular simulation results are already available in the payload database and can be retrieved directly, or whether MAGPIE is required to run the simulation with the provided data and parameter sets. To guarantee traceability, all resulting records will be deployed into the payload database via stored procedures. The *Shiny* server downloads the simulation data from MAGPIE and displays it.

### Data protection

#### Pseudonymization service

Pseudonymization adds an important layer of protection for person-related data [15]. We implemented a one-tier-pseudonymization via two separate databases: one for patient identifying data and one for pseudonymized medical (payload) data. At present, the two databases are only logically separated to simulate an operational environment with physical and spatial separation. The pseudonymization service is part of the application server and reunites pseudonymized medical data with patient identifying data as needed.

At the current prototype stage, we use anonymized patient data only. For demonstration purposes, e.g. to generate patient-specific predictions that can be used for individual treatment management, we complemented these *anonymized* data with artificial patient identifying information. In a later clinical application, a regulation-compliant pseudonymization service fulfilling the requirements of data protection needs to be implemented and complemented e.g. by a specific Trusted Third Party or another service as recommended by the Data Protection Working Group of the technology and method platform TMF e.V [16]. and in agreement with the Data Protection Officer at state and federal level.

#### Access control

A role-based access management system was developed to ensure that only authorized persons are allowed to access particular data. We defined permission objects (e.g. patient identifying data, core data, diagnostic data, treatment data, etc.) and user groups such as physicians, scientists, documentarists or administrator. Both are set into relation with defined access rights (read, update, create, delete).

#### Versioning control

For versioning control of payload data, we implemented an *insert-only* database. This means that users are not able to modify record sets directly in the database. If users execute the frontend’s *insert*, *update*, or *delete* actions, a new record set with “parent-child-information” for traceability will be inserted. Therefore, every modification is reproducible. Database views and stored procedures are provided to access current and historical data. This traceability is also established for model predictions (c.f [12].).

### Implemented mathematical disease models

In the described prototype, two mathematical models have been implemented for demonstration purposes. The framework itself is not restricted to these two particular models. It allows deployment of different mathematical models as long as they are registered in the MAGPIE model database, and feeding generated model predictions into the described workflow. There is no general restriction, neither on the model type nor on the particular implementation / programming language.

The single cell-based *CML model*, implemented in C++, describes both the pathogenesis and the standard treatment of chronic myeloid leukaemia patients ([5, 6, 8]). In brief, the clonal nature of the disease is seen as a competition between normal haematopoietic stem cells and a population of leukaemic stem cells. While the latter cells have a growth advantage in the untreated case, they are specifically targeted using tyrosine kinase inhibitor (TKI) therapy. As a result, the model reproduces the characteristic biphasic response pattern typically seen in CML patients. Adaptation of the model to individual time courses allows predictions about the patient’s future therapy response, in particular with respect to the expected long-term molecular response, measured clinically by BCR-ABL1 transcript levels in the peripheral blood.

The second example is a model that quantitatively describes *thrombopoiesis (*[17, 18]). It is part of a more general class of ordinary differential equation-based compartment models of human haematopoiesis ([2, 19]). These models consider haematopoietic stem cells, proliferating and maturing precursors, mature blood cells, as well as a number of growth-factor mediated feedback loops between these cell types. Respective pharmaceutical growth-factor applications and their pharmacokinetics and –dynamics are also considered as well as the effects of cytotoxic cancer therapy on proliferating cells and the bone marrow microenvironment. Predictions are generated for specifiable therapy options and at an individual patient level facilitating decision making in clinical practice. The model is implemented in R/Shiny calling C++ routines for improved numerical solving of the equations.

## Results

### Data management and exploration

**Fig. 2:**
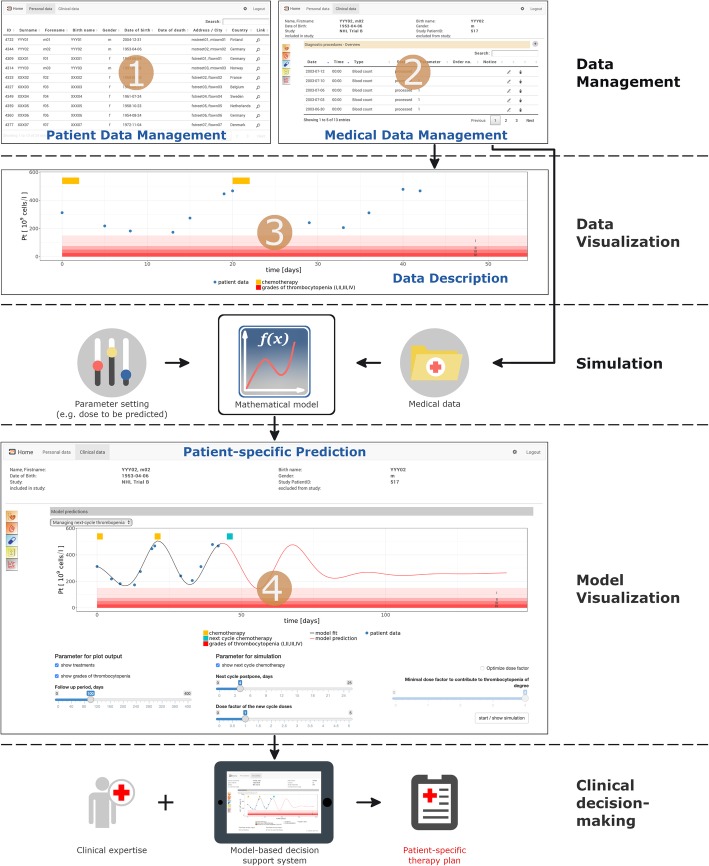
Schematic outline (screenshots) of framework components/features and information flows. Our prototype provides management of patient-identifying data (1) and corresponding medical data (2) complemented by an integrated graphical representation (3)*.* Mathematical model predictions can be generated interactively for user-defined parameter settings (slider-based parameter selection) and visualized in comparison to the clinical data (4). Supported by this integrated information, physicians are able to appraise different possible therapy scenarios and amendments for the treatment of individual patients (*Clinical decision-making*)

In order to support clinical decision-making for patient-specific therapy planning, our prototype unifies data management, data description in the form of visualizations, and patient-specific predictions based on mathematical disease models. Figure 2 illustrates corresponding features and information flows of our prototype software.

At the *Data management* layer, our framework supports the management of patient identifying data as well as of pseudonymized payload data including medical and core data. If a physician has the permission to access patient identifying data (see access control) the software allows retrieval of de-pseudonymized medical data. In contrast, any other user, such as a documentarist, modeler, system administrator, etc. has per default no access to patient identifying data. However, depending on the defined access rights, users are allowed to retrieve particular pseudonymized medical data. Furthermore, our framework provides access to diagnostic procedures, diagnoses, treatment information (e.g. details on drug types and dosing) or other evaluation data in pseudonymized form. Depending on permissions, users are allowed to add, modify, (soft)^2^ delete *core data*, *medical data*, and *patient identifying data* as explained in the section Access control.

To preserve data structure and to guarantee a high quality, we designed predefined data entry forms. However, to allow for flexibility, these can be customized by users on the basis of *core data* definitions. The *core data* sets define authorized entries of *medical* and (within pre-defined access rules) *patient identifying data*. As an example, when inserting leukocyte values from the peripheral blood for the first time, one has to define the core data “blood count” as screening type, “leukocyte” as diagnostic parameter, and the measurement unit, e.g. “10^9^/l”. Thereafter, these entries are available in drop-down fields for insertion and editing. Additional information (i.e., metadata), such as date and time, screening status, order no., etc. are added automatically by the system or can be added in a user-defined way.

For the visual data description/exploration, we apply interactive *Shiny* routines. Visualization of monitoring parameters, e.g. time courses of leukocyte or platelet counts, or the proportion of Philadelphia-positive cells as well as BCR-ABL1 transcript levels, can (optionally) be presented together with therapy details and reference values/ranges. These visualizations help physicians to get a faster and more detailed overview of therapy conditions and corresponding patient responses (see Fig. 2: Data management / Data visualization).

### Model-based decision support

On top of the data management and visualization features, our framework provides predictions, generated by mathematical models or computer simulations, to aid the physician’s decision making by complementing medical data with another level of information. The availability of model predictions might also help to communicate therapeutic decisions or potential alternative treatment scenarios to the patient and, therefore, to improve treatment compliance.

Based on the structured and visually presented clinical data, the physician can identify uncertainties or open questions that hamper a clear-cut therapeutic decision, such as expected patient-specific toxicity of treatment or necessity of individual treatment adaptations according to expected response. Such questions can then be addressed e.g. by simulating different treatment options for the same patient and generating corresponding model predictions “on-the-fly”. With the presented framework, the physician can specify the parameter settings (e.g. potential drug dose amendments, alternative application schedules or prediction intervals) directly within the GUI of the data management platform. Once the parameters have been selected, a simulation run can be started (Fig. 2: *Simulation*) and the patient-specific predictions are graphically presented together with the corresponding clinical data (Fig. 2: *Model Visualization*). The physician can appraise and potentially use this integrated information (data + model prediction) to arrive at his/her therapeutic decision.

It is also possible to generate model predictions for a spectrum of different parameter settings (e.g. a range of potential drug doses) to study potential effects sizes or sensitivities of expected patient-specific responses. All these “virtual treatments” (i.e. the MAGPIE project/job IDs of these particular simulations and the corresponding simulation data) are managed in the payload database (see Fig. 1) and are therefore always and completely reproducible.

### Example applications / use cases

To demonstrate the functionality of our framework as a model-based clinical decision support system, we present two prototypic applications.

#### Prediction of treatment-response dynamics in CML (use case 1)

**Fig. 3:**
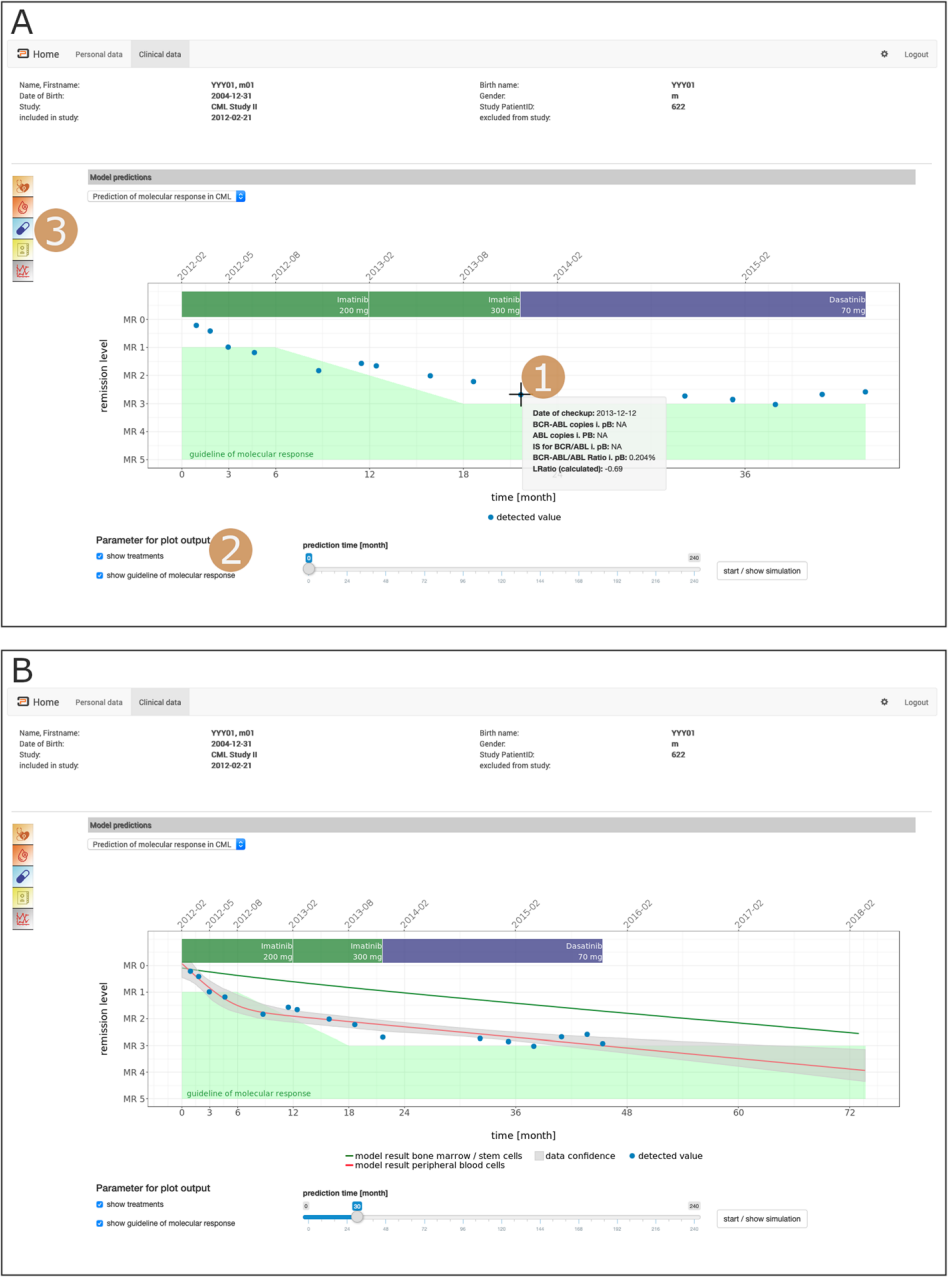
Screenshots illustrating the presentation of patient-specific TKI-treatment response dynamics in CML. Patient-identifying data (name, birth data etc.) have been changed to artificial values to ensure anonymity. **A**) Annotated graphical representation of data. 1) Visualization of BCR-ABL1 levels, i.e. molecular response in the peripheral blood (blue dots). 2) This information can be optionally complemented by further therapy details, i.e. TKI type / dose (coloured / annotated bar on top of diagram) or clinical target levels, e.g. as suggested by clinical guidelines (green shaded area). 3) Menu for accessing further patient-specific clinical information, e.g. further diagnostic parameters, therapies, diagnoses. **B**) Data as shown in panel A, complemented by model predictions for BCR-ABL1 levels in peripheral blood (red line) with corresponding pointwise 95% confidence intervals and by predicted remission levels of leukaemic stem cells in the bone marrow (green line). The latter prediction relates to a cell cycle inactive (“TKI-protected”) subpopulation of leukaemic stem cells

First, we implemented an example to illustrate decision support for CML patients under continuous tyrosine kinase inhibitor (TKI) therapy. In this disease, the level of the aberrant BCR-ABL1 mRNA in the peripheral blood provides a surrogate measure for tumour/leukaemia load, and is in routine clinical use for monitoring the patient’s treatment response ([8, 20, 21]). Our software visualizes the BCR-ABL1 level over time, and allows annotation of the raw data points by different reference values and/or actual treatment modalities, such as TKI type/dose (Fig. 3a). Targeting the individual data point with the mouse cursor will provide additional information about this particular measurement, e.g. quality criteria such as the underlying copy number or whether this data points has been standardized on the international scale (IS).

Applying our established mathematical CML model ([5, 8]) to an individual patient time course (i.e., estimating the model parameters from BCR-ABL1 measurements) allows to derive and visualise patient specific predictions about the expected future treatment response (Fig. 3b). Especially, the estimated abundance of residual leukemic stem cells is not accessible in the clinic and provides additional model-derived information, e.g. in the case that treatment cessation is considered. Currently, the CML model provided assumes a fixed TKI dose. For mathematical detailed about the modelling and the parameter estimation, we refer to [8].

Additionally to the BCR-ABL1 levels together with the model predictions, the user is able to access further clinical parameters that are available for this patient. These can be retrieved and visualized via the graphical menu (c.f. Fig. 3a).

#### Prediction of thrombocytopenia under cytotoxic chemotherapy (use case 2)

As a second illustrative example, we implemented the individualized mathematical model of human thrombopoiesis, applied to patients with aggressive NHL treated with six cycles of a combination therapy of four to five cytotoxic drugs, i.e. applying the CHOP / CHOEP chemotherapy regiments studied in [22]. In these protocols, cycle duration is either 14 or 21 days. Patients treated with these chemotherapies are at high risk for developing life-threatening haematotoxicity during the course of the therapy [23]. Predicting which of the patients suffer these severe conditions is of high clinical relevance to take countermeasures such as prophylactic hospital stay, postponement of therapy or reduction of chemotherapy dosage.

The mathematical thrombopoiesis model ([17]) uses individual platelet time course data and the applied therapy schedules together with population data from the literature to estimate individual model parameters. These parameters can be used to predict individual future platelet counts of this particular patient. This includes simulations of treatment adaptations, aiming to minimize thrombocytopenia while maintaining sufficient treatment efficacy.

**Fig. 4:**
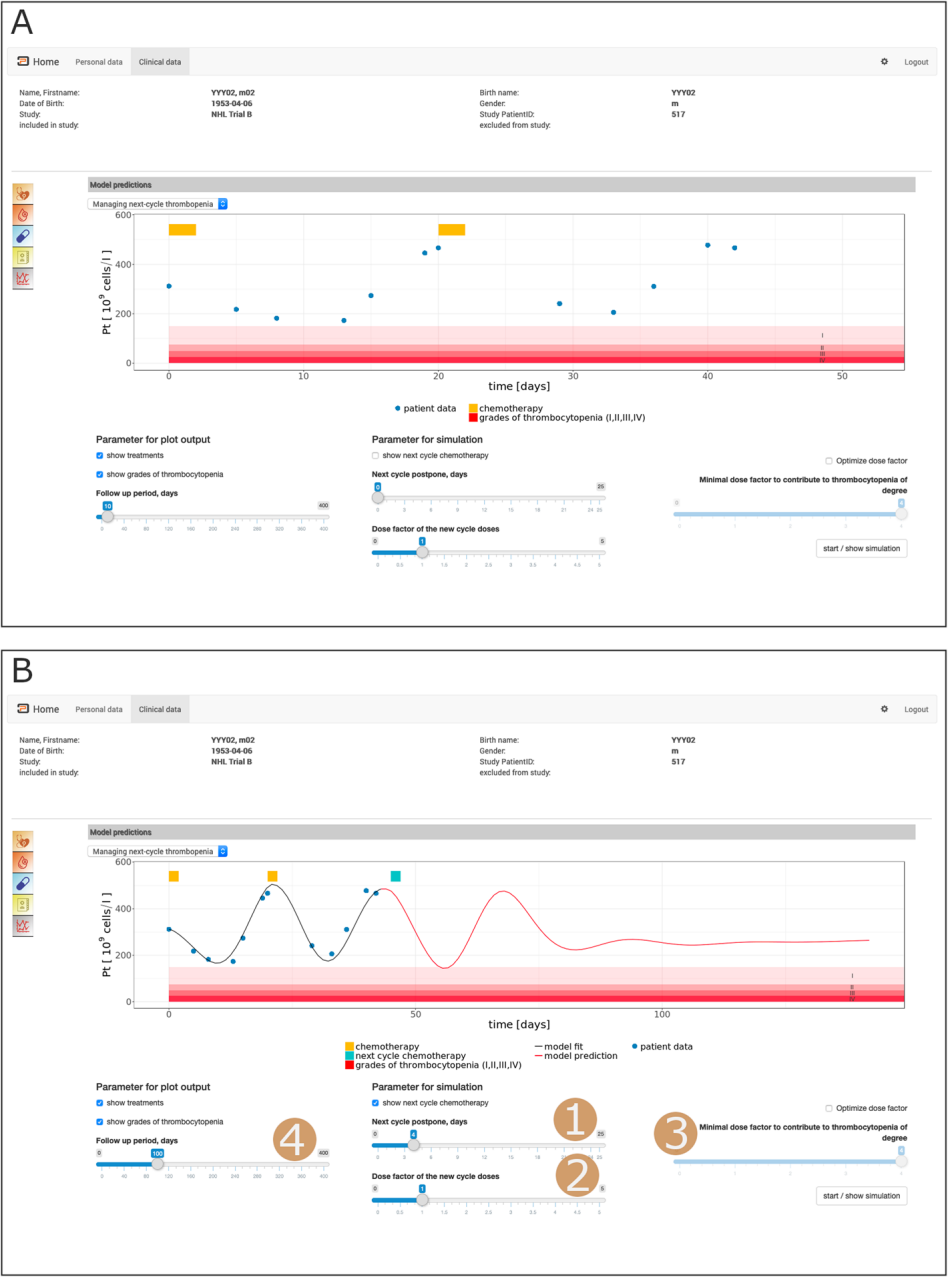
Screenshots illustrating the presentation of patient-specific chemotherapy-induced side-effects on thrombopoiesis. Patient-identifying data (name, birth data etc.) have been changed to artificial values to ensure anonymity. **A**) Presentation of platelet dynamics of a single NHL patient and corresponding therapy schedule. Days with chemotherapy applications are marked by orange bars. Degrees of thrombocytopenia (red-shaded areas) can be optionally displayed. Further available patient-specific clinical parameters can be assessed via the GUI menu (c.f. Fig. 3a) **B**) Visualization of model fit for the observed data and model prediction for the next chemotherapy cycle for a use-defined treatment scenario. Possible options for treatment adaptations are: 1) Postponement of the next cycle, 2) Factor for dose adaptation (1 = no change), 3) Dose factor required to tune toxicity to a tolerable limit. The follow-up duration to be simulated can be also modified (4). Continuation of the previously applied dose with 4 days postponement and a prediction period of 100 days

The simulations can be configured by selecting treatment options such as dosing of drugs. Results are visualized within the GUI of our framework (Fig. 4). In brief, available clinical data of a specific patient, including basic patient characteristics, planned treatment protocol and platelet counts during therapy can be directly assessed and visualized (Fig. 4a). Boundaries of the different degrees of thrombocytopenia are also provided, if desired by the user. So far, available platelet counts and prior data are used to derive individual parameter estimates. Based on this parameter set, the user can perform simulations of future treatments including adaptations of the originally planned protocol. For this purpose, the start of the next therapy cycle can be shifted by a specified number of days. Moreover, doses of all cytotoxic drugs can be adapted, or the software determines a dose factor to tune the degree of thrombocytopenia to a tolerable level. Corresponding model predictions can be displayed for a specified follow-up time together with the currently available data (Fig. 4b). This model-based assessment of different treatment options supports clinical decision-making regarding timing and dosing of the next therapy cycle.

## Discussion

We present a framework to support diagnostic and therapeutic decision-making in haematology based on patient specific time course data and individualized mathematical model predictions. Using a prototype implementation, we demonstrate in a *proof-of-principle* manner how systems medical (i.e. theoretical and/or computational) methods can be integrated into clinical practice. In contrast to other published clinical decision support (CDS) frameworks ([24–26]), we focus on complementing existing workflows and data management environments that are familiar to clinical users by patient-specific model predictions and, therefore, to allow for easy and straight forward application of systems medical tools.

Our framework follows a strictly modular structure. That means that all its components (i.e., the GUI, the database(s), the pseudonymization service, the application servers, and the mathematical models itself) are independent and, therefore, exchangeable. Specifically, the integration of model predictions (i.e., the model server functionalities) including their graphical representation could in principle be integrated into any existing clinical data management software, e.g. by using REpresentational State Transfer Application Programming Interfaces (REST APIs). Although not yet implemented, such extensions are straightforward. In order to allow for a future more rigorous software development process (e.g. to generate a certificated medical device), we prepared standard operating procedures (SOPs) to make the current software design and development transparent and comprehensible.

An additional degree of flexibility results from the integration of the versatile MAGPIE model server. As this server is designed to work with virtually any type of model, irrespective of particular implementation (i.e. the programming language) [12], no general restrictions regarding the language in which the model is implemented are necessary. For example, our thrombopoiesis model (use case 1) is implemented in R while our CML model (use case 2) is implemented in C++. Likewise, statistical models and pipelines, such as regression models, classification algorithms or other statistical learning procedures can also be integrated into the MAGPIE environment. Also, with respect to the endpoint or the clincial question, different models could be provided. Whereas the current prototype version of the framework includes just one predictive model for each of the two example diseases, this is not a general restriction and a selection of different models for the same disease could be provided to the user. Clearly, validity of the provided models have to be tested and guranteed. Furthermore, the particular parametrization options provided to the user for each of these models have to be carefully selected to allow for easy handling in the particular clinical situation.

The access time of individual model predictions determines the usability of our application in clinical practice. This time is largely defined by the requirements for the numerical model calculations. Whereas fairly simple ODE-based model predictions are available within seconds, more extensive single-cell based approaches, involving several rounds of optimization, could potentially result in simulation times of several hours. While a “real-time” bedside evaluation might be possible in the first case, the latter case might require a database of already pre-performed simulations. In order to cope with this issue, we established a job versioning to allow easy access to available simulations. This way, patient-specific predictions can be generated at any time new data becomes available (e.g. by overnight batch processing), stored in the database, and immediately accessed if needed.

There are an increasing number of publications, also describing computational tools for clinical decision-support. While knowledge bank approaches provide clinically relevant information in a comprehensive format (e.g. [27–29]), clinical decision-support systems additionally provide personalized predictions based on statistical / evidence-based models (e.g. [24, 25]). A workflow-driven approach presented by Bucur et al. [26] is of particular interest in comparison to our approach, as it focuses on the integration of different types of knowledge models into the process of evaluating and defining interdisciplinary therapy plans. While this approach also integrates predictions based on functional dynamical models, it concentrates on generating new workflows across several phases of individual patient care, such as data review, diagnosis, and treatment selection. In contrast, our framework focuses on the integration of model predictions into existing workflows and data management systems, with the key objective to lowering the barriers for using computational models and simulations in a clinical “real-world” setting. Furthermore, our approach has specifically been designed to allow for the use of computational models in clinical settings (i.e. clinical trials and routine use) by ensuring a high level of transparency and traceability. Specifically, our framework provides a complete audit trial functionality not only for clinical data but also for model code, simulation runs, parameter settings and individual model predictions.

The presented framework has been tested by different project partners, all with a background in haematology and/ or oncology. Their feedback, regarding practical relevance and usability went directly into the presented implementation. Also, we initiated a so called “virtual trial”, which is accessing the acceptance of model predictions provided within a general data management environment in the context of clinical decision-making. In this (still ongoing) study clinicians from different hospitals and cancer centres outside our consortium are involved as test users. The implemented models itself as well as the MAGPIE model server have already been tested and validated independently ([1–8, 12]). Although tested for model correctness and for general usability, the presented framework is still a prototype. To be applied in clinical routine, in particular the pseudonymization service still needs to be implemented and the access control will have to be extended in order to allow for a save and regulation conform application.

## Conclusion

We present a biomedical informatics approach to facilitate the utility of systems medical models to support decision-making in clinical practice. This is achieved by combining data management, presentation and exploration, and most importantly, user-specifiable model simulations of treatment options at an individual level and presentation of the results in an easily interpretable fashion. By integrating mathematical model predictions in a transparent and save manner directly into established clinical workflows, our framework can considerably foster the translation of systems-medical approaches into practice. We illustrated this by two working examples from the field of haematology / oncology.

## Availability and requirements

Project name: HaematoOPT Demonstrator.

Project home page: https://hopt.imb.medizin.tu-dresden.de

Operating systems: client side: platform independent; server side: Microsoft Windows Server 2008 R2 for database server, Ubuntu 16.04.2 LTS for application, visualization server and model server

Programming language: PHP 7, R

Other requirements: Microsoft SQL Server 2008 R2, Apache 2.4.18, MAGPIE

License: Creative Commons BY license (for own code; does not apply for MS Windows / SQL Server).

Any restrictions to use by non-academics: no.

## Supplementary information

**Additional file 1:** List of necessary software features.

**Additional file 2:** Entity Relationship Model (ERM).

## Data Availability

A demo server can be accessed at https://hopt.imb.medizin.tu-dresden.de. Electronic supplementary materials are listed below: additional-file-1.pdf Additional file 1: List of necessary software features. additional-file-2.pdf Additional file 2: Entity Relationship Model (ERM). additional-file-3.mp4 Additional file 3: Demo server video tutorial.

## Footnotes

1 According to “ISO Technical Specification 25,237 – Health informatics – Pseudonymization”, the term *payload* is used for all other data that are not identifying.

2 Instead of actually deleting a record, it is flagged as deleted
