# Supplementary material for: Integration of mathematical model predictions into routine workflows to support clinical decision making in haematology
Source: BMC Med Inform Decis Mak. 2020 Feb 10;20:28. doi: 10.1186/s12911-020-1039-x (PMC7011438; doi:10.1186/s12911-020-1039-x)
Supplement: Supplementary file 2 — Additional file 1: List of necessary software features. [file 12911_2020_1039_MOESM1_ESM.pdf]

Integration of mathematical model predictions into routine workflows to support clinical decision making in haematology

Additional file 1 - List of necessary software features

Authors:

Katja Hoffmann[1], Katja Tampe[1], Christoph Baldow[1], Silvio Schuster[1], Yuri Kheifetz[2], Sibylle Schirm[2], Matthias Horn[2], Thomas Ernst[3], Constanze Volgmann[3], Christian Thiede[4], Andreas Hochhaus[3], Martin Bornhäuser[4,6], Meinolf Suttorp[5], Markus Scholz[2], Ingmar Glauche[1], Markus Loeffler[2], Ingo Roeder[1,6]

[1] Institute for Medical Informatics and Biometry, Faculty of Medicine Carl Gustave Carus, Technische Universität Dresden, Dresden, Germany

[2] Institute for Medical Informatics, Statistics and Epidemiology, Faculty of Medicine, University of Leipzig, Leipzig, Germany

[3] Abteilung Hämatologie/Onkologie, Klinik für Innere Medizin II, Universitätsklinikum Jena, Jena, Germany

[4] Department of Internal Medicine, Medical Clinic I, University Hospital Carl Gustav Carus Dresden, Dresden, Germany

[5] Pediatric Hematology and Oncology, Department of Pediatrics, University Hospital Carl Gustav Carus Dresden, Dresden, Germany

[6] National Center for Tumor Diseases (NCT), Partner Site Dresden, Dresden, Germany

The following list enumerates the necessary software features of the presented web application.

1. Only authorized persons will be able to access to the database application.

2. Only authorized persons will be able to access patient identifying data in dependency of their permissions.

3. The software needs to provide the functionality to define user permissions. This needs to be implement as a role-based user access control.

4. Medical data has to be stored/kept separately from identifying patient data (Pseudonymization).

5. There has to be a functionality to reconnect medical and simulation data to recover patient identifying information (De-pseudonymization).

6. Authorized users should be able to search for patients by identifying data (e.g. Name, Date of birth) or pseudonymized data (e.g. global or study internal patient identifier).

7. Following pseudonymized data should be managed by the software.

   - Diagnostic procedures with diagnostic parameters
   - Diagnoses and other results from clinical examinations
   - Treatments
   - Any other freely definable patient data

8. Authorized users should also be able to add, modify or delete data.

9. The software / database design should be developed such that different studies with different clinical data can be handled.

10. Redundancies have to be avoided.

11. The data quality has to be ensured by defining global master data. This includes the used-based definition of e.g. trial information, diagnostic parameters, drugs, units.

12. The application has to be implemented with an audit trail / data version control system to trace every data modification.

13. The application needs to provide the following data visualizations:

    - time course for BCR-ABL1 transcript levels with treatment details for patients with CML.
    - Platelet dynamics with treatment details for NHL patients.

14. The application needs to integrate different computer models by interacting with approbriate model servers. The following model-based predictions need to be included in the demo server:

    - prediction of treatment-response dynamics in patients with CML
    - prediction of thrombocytopenia under cytotoxic chemotherapy for NHL patients

15. Simulation calls and results from model simulations need to be stored and recalled, if necessary. Patient specific model simulations shall only be initiated if there are any updates in the medical data or changes in the parameter sets.

16. The user interface should be easy and intuitive to use.
