# Supplementary material for: Integration of mathematical model predictions into routine workflows to support clinical decision making in haematology
Source: BMC Med Inform Decis Mak. 2020 Feb 10;20:28. doi: 10.1186/s12911-020-1039-x (PMC7011438; doi:10.1186/s12911-020-1039-x)
Supplement: Supplementary file 3 — Additional file 2: Entity Relationship Model (ERM). [file 12911_2020_1039_MOESM2_ESM.pdf]

Integration of mathematical model predictions into routine workflows to support clinical decision making in haematology

Additional file 2 - Entity Relationship Model (ERM)

Authors:

Katja Hoffmann[1], Katja Tampe[1], Christoph Baldow[1], Silvio Schuster[1], Yuri Kheifetz[2], Sibylle Schirm[2], Matthias Horn[2], Thomas Ernst[3], Constanze Volgmann[3], Christian Thiede[4], Andreas Hochhaus[3], Martin Bornhäuser[4,6], Meinolf Suttorp[5], Markus Scholz[2], Ingmar Glauche[1], Markus Loeffler[2], Ingo Roeder[1,6]

[1] Institute for Medical Informatics and Biometry, Faculty of Medicine Carl Gustave Carus, Technische Universität Dresden, Dresden, Germany

[2] Institute for Medical Informatics, Statistics and Epidemiology, Faculty of Medicine, University of Leipzig, Leipzig, Germany

[3] Abteilung Hämatologie/Onkologie, Klinik für Innere Medizin II, Universitätsklinikum Jena, Jena, Germany

[4] Department of Internal Medicine, Medical Clinic I, University Hospital Carl Gustav Carus Dresden, Dresden, Germany

[5] Pediatric Hematology and Oncology, Department of Pediatrics, University Hospital Carl Gustav Carus Dresden, Dresden, Germany

[6] National Center for Tumor Diseases (NCT), Partner Site Dresden, Dresden, Germany

Table of Content

Table of Content 2
1 Introduction 2
1.1 Purpose 2
1.2 Glossary 2
2 ERDs of Identifying patient database 2
2.1 Patient identifying data 2
3 ERDs of pseudonymized payload database 3
3.1 Pseudonymized patient data 3
3.2 Medical data 4
3.2.1 Diagnostic procedures 4
3.2.2 Treatments 5
3.2.3 Evaluation 7
3.2.4 Side Effects 7
3.2.5 Any further patient data 8
3.3 Simulation data 9
3.4 Access management 10
4 Traceability of data inserts, updates and deletes 11

1 Introduction

1.1 Purpose

The purpose of this document is to present the entity relationship model for the demo server https://hopt.imb.medizin.tu-dresden.de via entity relationship diagrams. It will explain the entity types and specifies relationships that can exist between the instances of those entity types.

1.2 Glossary

**Term**	**Definition**
ERD	Entity Relationship Diagram
ERM	Entity Relationship Model
FK	Foreign key
N	Null
pB	Peripheral blood
STC	Stem cell transplantation
UK	Unique key

2 ERDs of Identifying patient database

2.1 Patient identifying data

Fig. 1 shows the ERD for storing patient identifying data. The table *PatientPersonalData* contains current and *PatientPersonalDataHist* contains historical patient identifying data. The column PatientID of the table *PatientPersonalData* uniquely identifies each patient. The column PID of table *PatientPersonalData* is also a unique identifier for patient and is used for merging medical data of pseudonymized payload database and patient identifying data (compare table Patients in Fig. 2).

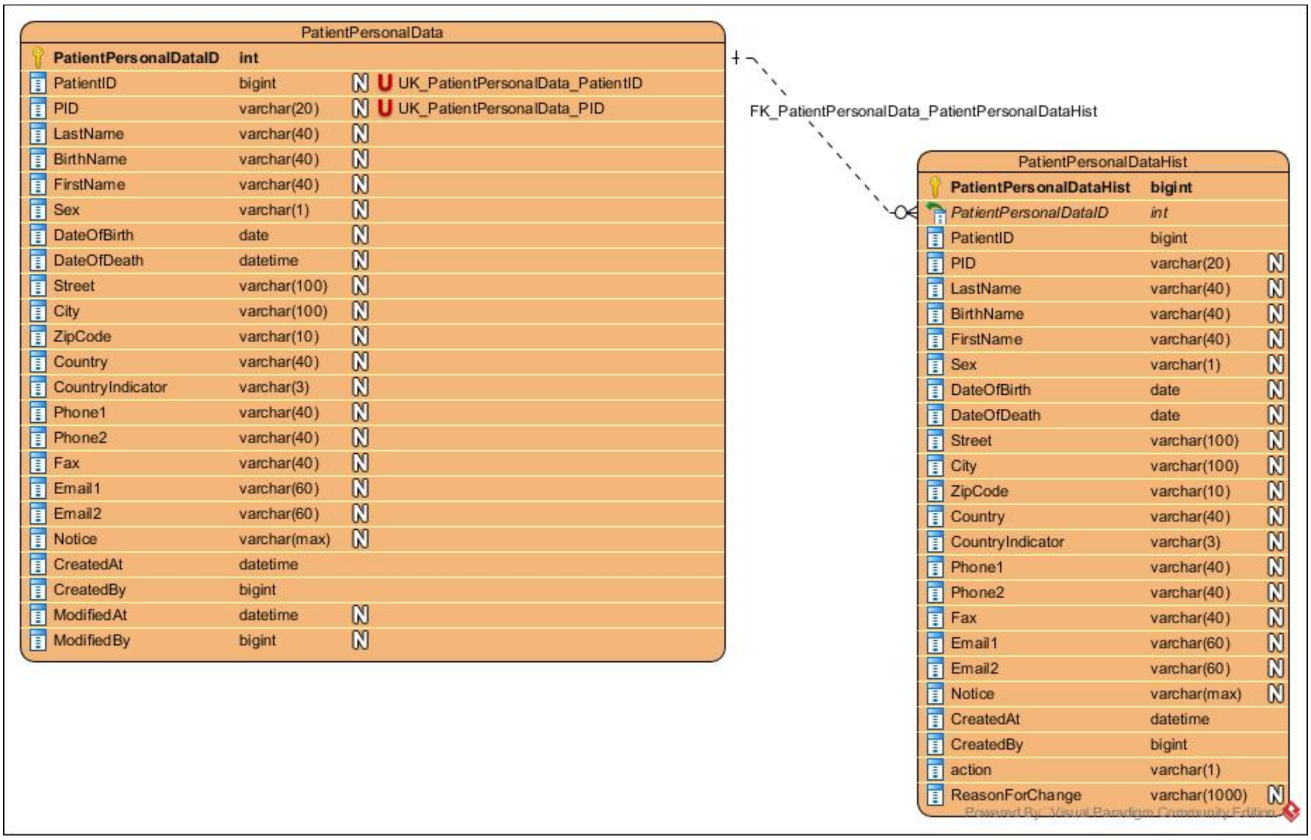

Fig. 1: ERD for management of patient identifying data

3 ERDs of pseudonymized payload database

3.1 Pseudonymized patient data

Fig. 2 shows the ERD for management of studies / trials (table *Studies*) and their study participants (table *PatientStudies*).

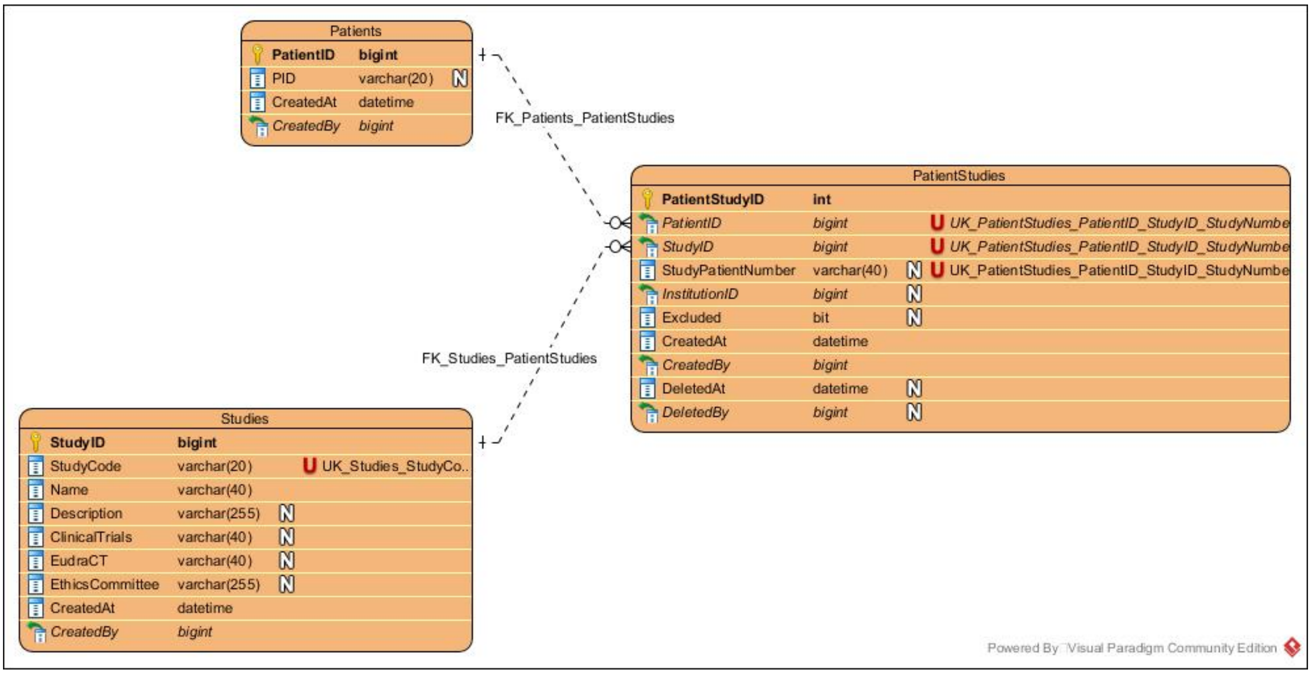

Fig. 2: ERD for management of studies / trials and their participants (patients)

The management of relevant institutions and their contact persons is already prepared (see Fig. 3) but not yet implemented in the presented web application.

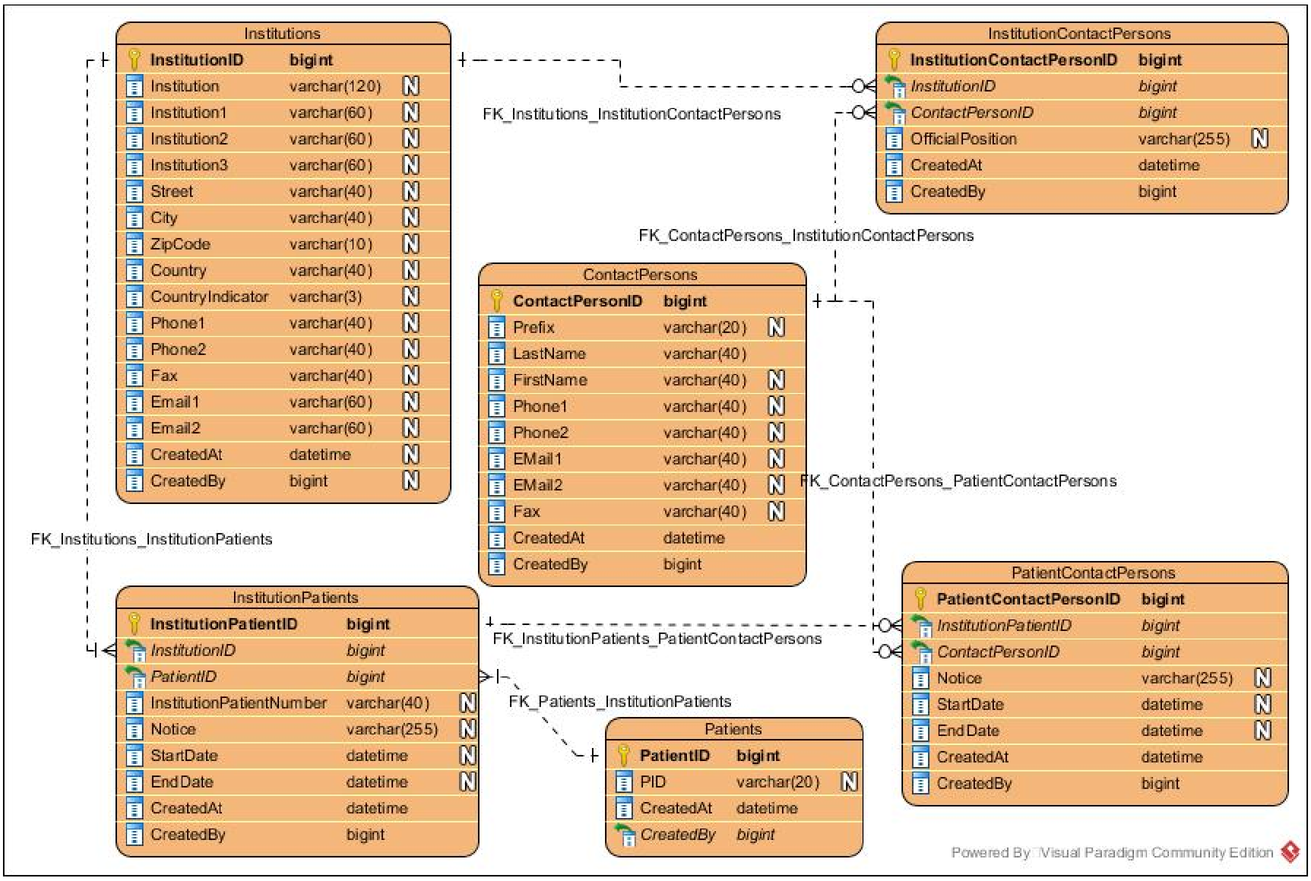

Fig. 3: ERD for management of institutions and contact persons

3.2 Medical data

3.2.1 Diagnostic procedures

Fig. 3 depicts the ERD for management of diagnostic procedures. The table *Checkups* contains each diagnostic procedure per row and the table *CheckupValues* contains each diagnostic value per row. These tables contain current as well as historical record sets that are managed by the attributes VersionPrev, VersionPost and ReleaseStatus, see section Traceability of data inserts, updates and deletes.

The tables *CheckupsTypes*, *CheckupParameters*, *CheckupParameterCategories* and *Units* are master data. The table *CheckupTypes* contains each type of visit (e.g. blood count, hormone test). The table *CheckupParameters* contains each diagnostic parameter per row (e.g. leukocyte i. pB, thrombocyte i. pB, BCR-ABL1 i. pB). The table *CheckupParameter-Categories* contains the category options for each diagnostic parameter.

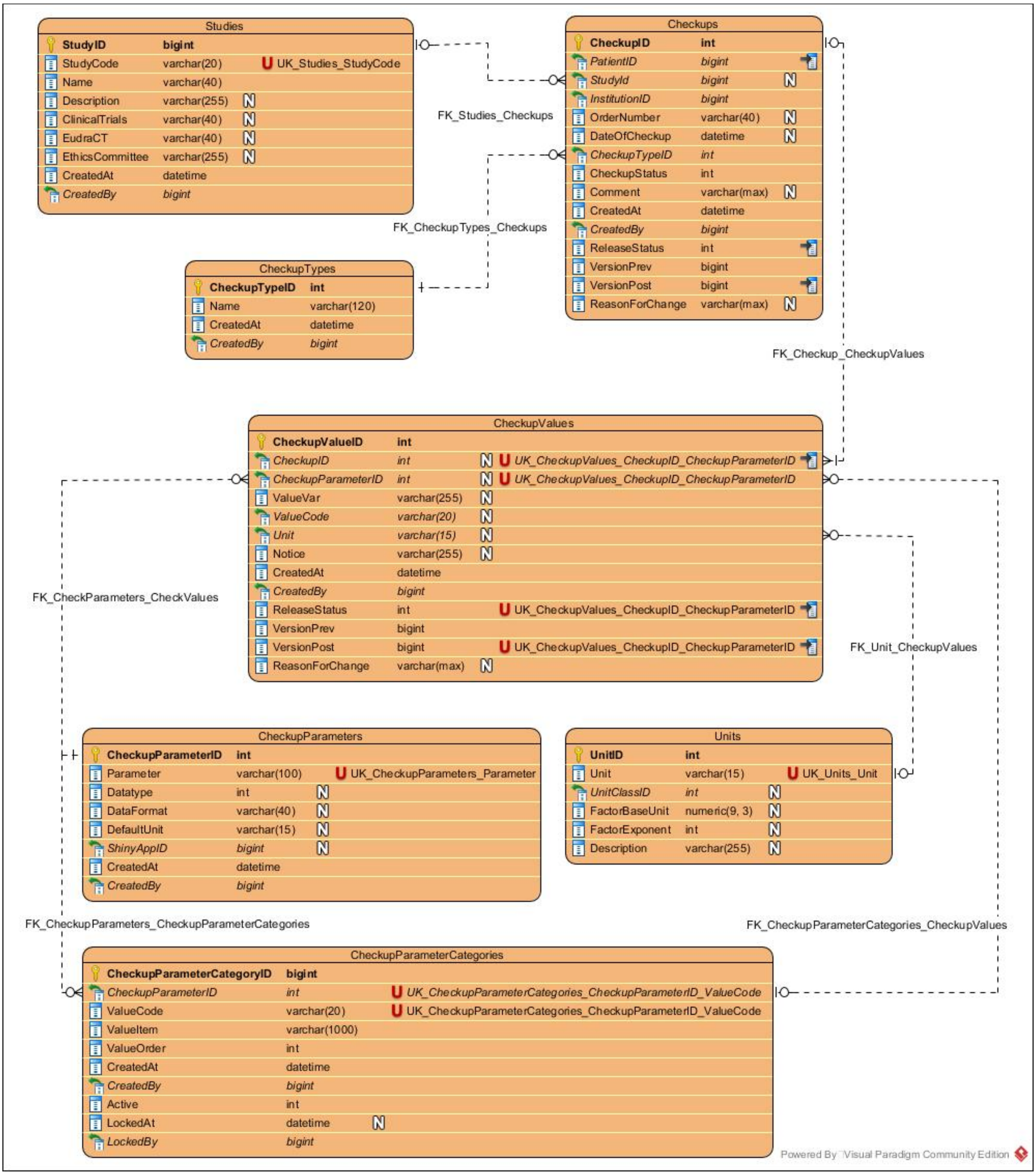

Fig. 4: ERD for management of diagnostic procedures

3.2.2 Treatments

Fig. 4 depicts the ERD for management of treatments. The table *Treatments* contains each treatment per row. The table *TreatmentValues* contains each treatment value per row if treatment type is not a drug administration, comp. column "ValueTable" of table *TreatmentType*. Information of drug administration are stored in table *TreatmentDrugAdministrations*. *Treatments*, *TreatmentValues* and *TreatmentDrugAdministrations* contain current as well as historical record sets that are managed by the attributes VersionPrev, VersionPost and ReleaseStatus, see section Traceability of data inserts, updates and deletes.

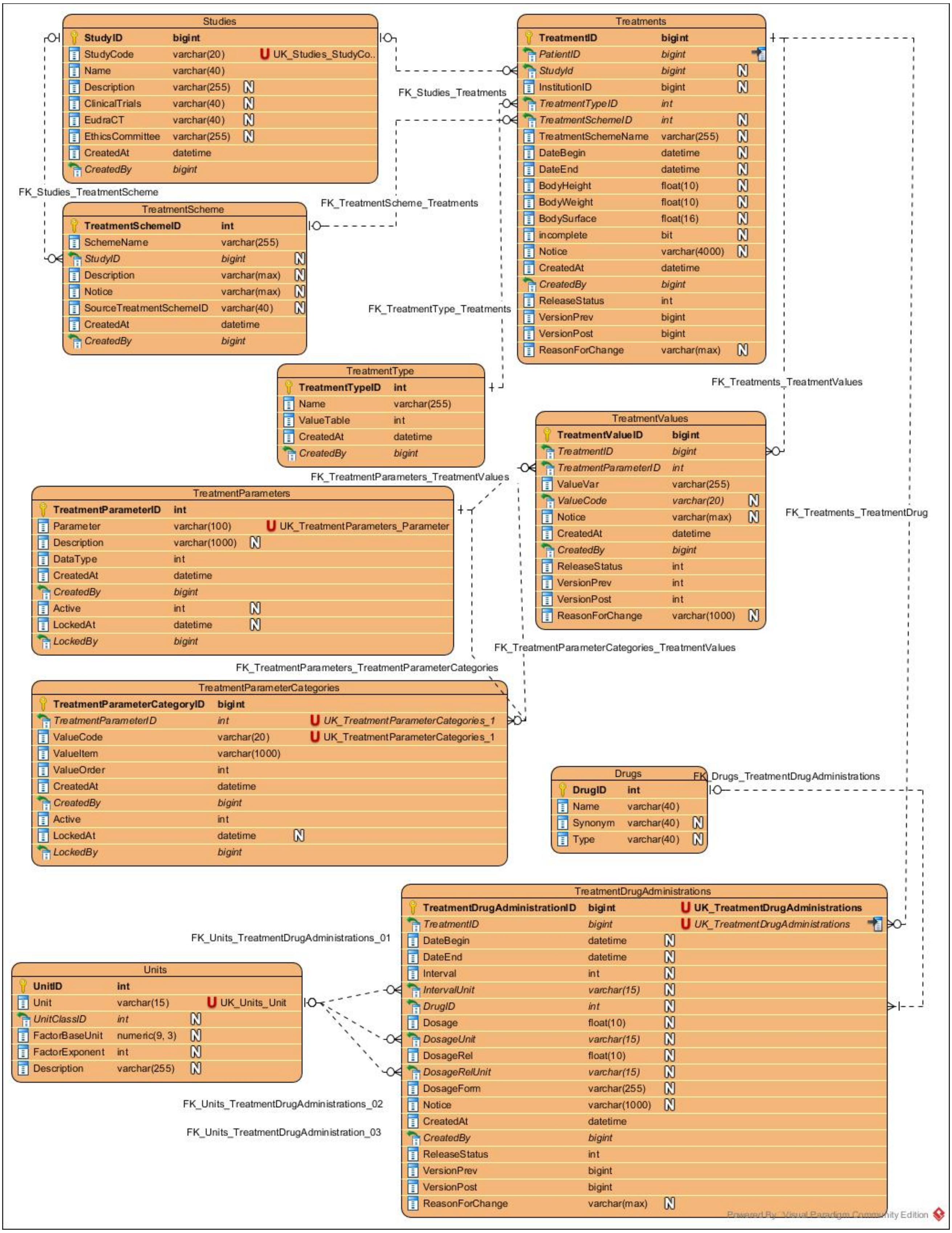

Fig. 5: ERD for management of treatments

The data in tables *TreatmentType*, *TreatmentScheme*, *TreatmentParameters*, *TreatmentParameterCategories*, *Drugs* and *Units* are master data. *TreatmentType* contains each type of treatment per row (e.g. chemotherapy, stem cell transplantation). The table *TreatmentScheme* contains each drug administration scheme per row (e.g. CHOP14, CHOEP21). The table *TreatmentParameters* contains parameters with options that are stored in table *TreatmentParameterCategories* (e.g. SCT type with the options allogeneic and autologous). The table *Drugs* contains each drug per row and the table *Units* contains each unit per row.

3.2.3 Evaluation

Fig. 5 depicts the ERD for management of diagnoses and any other evaluation. The table *Evaluation* contains each diagnose or evaluation per row. Current and historical record sets are managed by the attributes VersionPrev, VersionPost and ReleaseStatus, see section Traceability of data inserts, updates and deletes.

The table *EvaluationItems* contains each evaluation parameter per row and the table *EvaluationCategories* contains predefined options for the parameter.(e.g. for the evaluation parameter “CML phase” exists the options “chronic phase”, “accelerated phase” and “blast crisis phase” that can be used as evaluation value in table *Evaluation*.

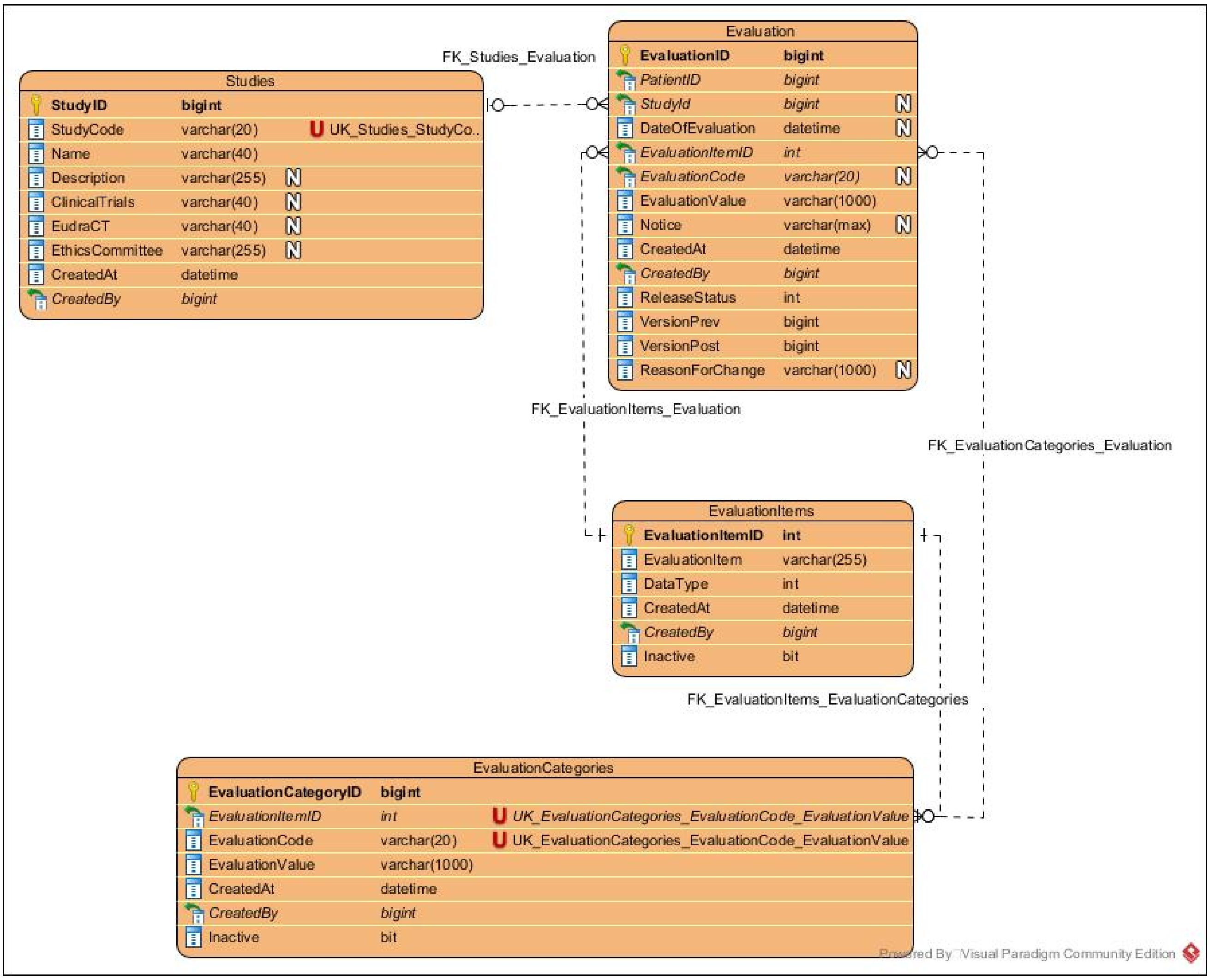

Fig. 6: ERD for management of evaluation

3.2.4 Side Effects

The management of records on side effects is not yet implemented in the current version of the application, although the necessary features are already prepared, see Fig. 7. Adverse events and their grades of intensity are managed as master data via tables *AdverseEvents* and *AdverseEventIntensity*. Side effect data are stored in table *AdverseEffects* and their relationship to a treatment are stored in table *AdverseEffectsTreatments*.

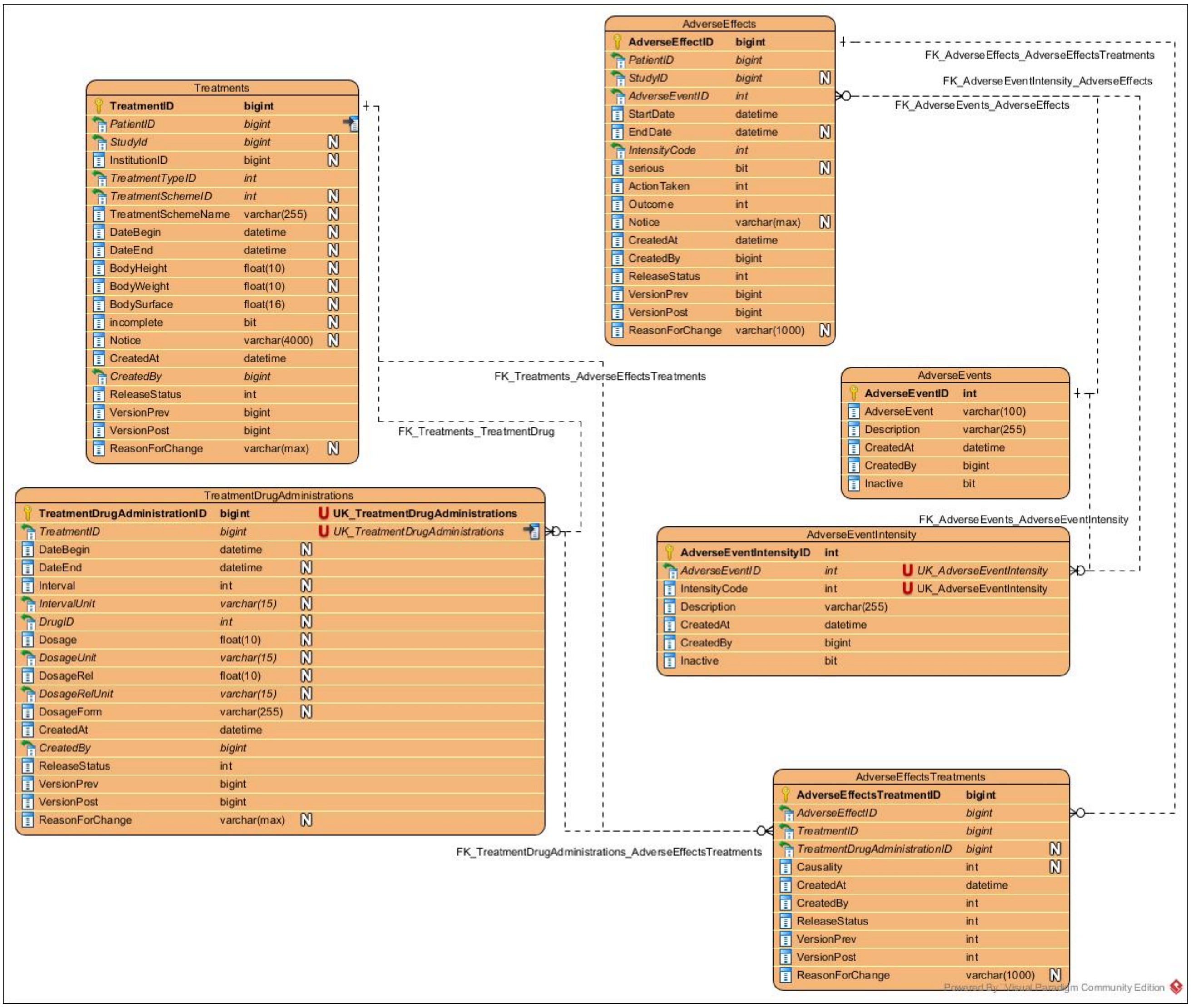

Fig. 7: ERD for management of side effects

3.2.5 Any further patient data

Any further patient data that are not diagnostic values, treatments, evaluation or side effects (e.g. gender, trial entry age, trial specific stratifications) are stored in table *PatientDataValues*. The parameters and their categories as well as the study affiliation are managed as master data in tables *PatientDataItems*, *PatientDataItemCategories* and *Patient-DataItemStudies*, see Fig. 6.

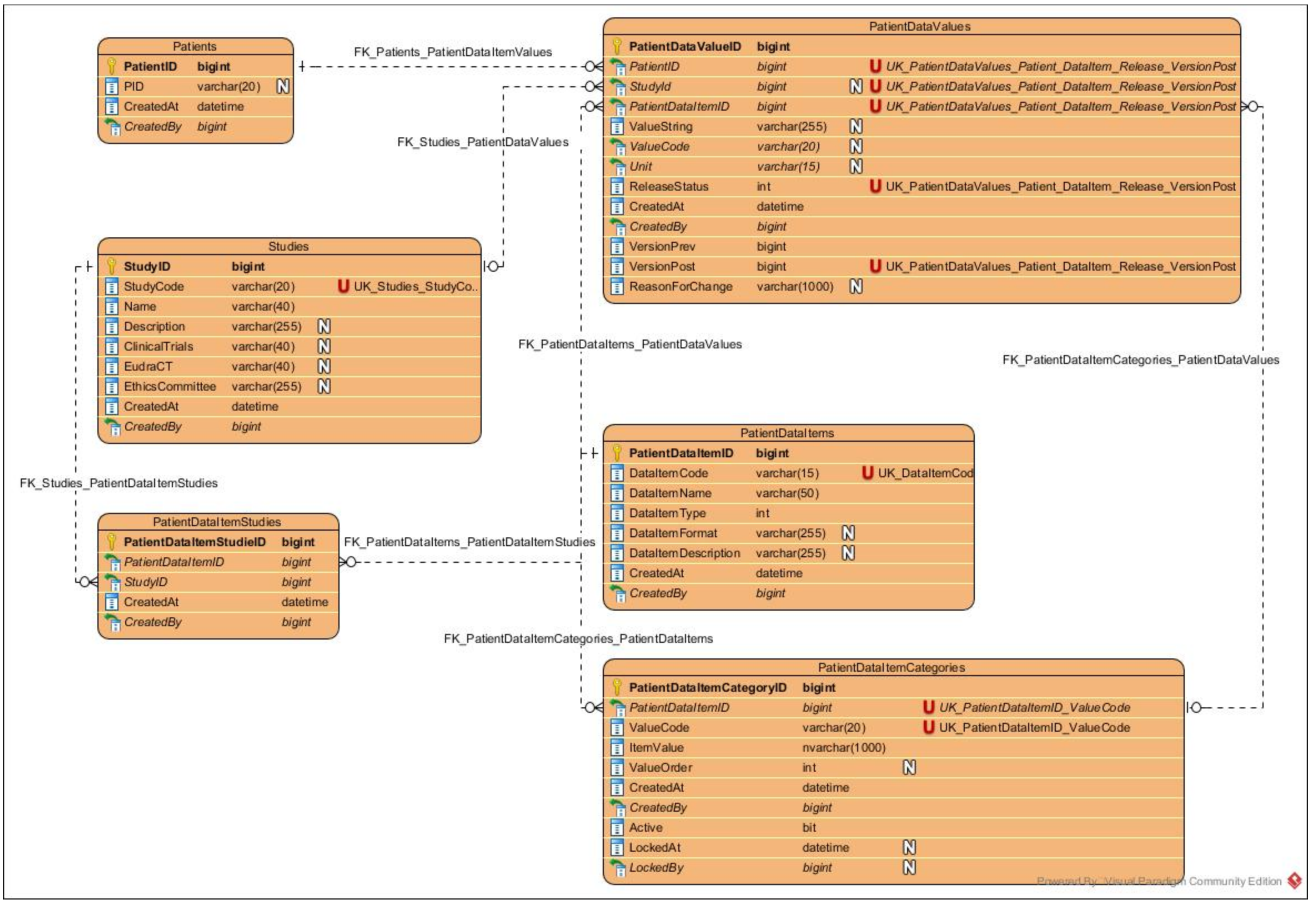

Fig. 8: ERD for management of any other data that are not diagnostic values, treatments, evaluation or side effects

3.3 Simulation data

Fig. 9 shows the ERD for management of simulation data. The table *MagpieRuns* contains each model prediction per row with the MAGPIE project ID, job ID and the used parameter set. The table *SQLStatement* contains every used SQL Statement to query current and historical datasets. With every simulation run the database version is saved in the table *SQLQueries* via the last primary keys of the tables that contains patient data (comp. columns Patient-DataValueMAX till AdverseEffectMax of table *SQLQueries* in Fig. 9).

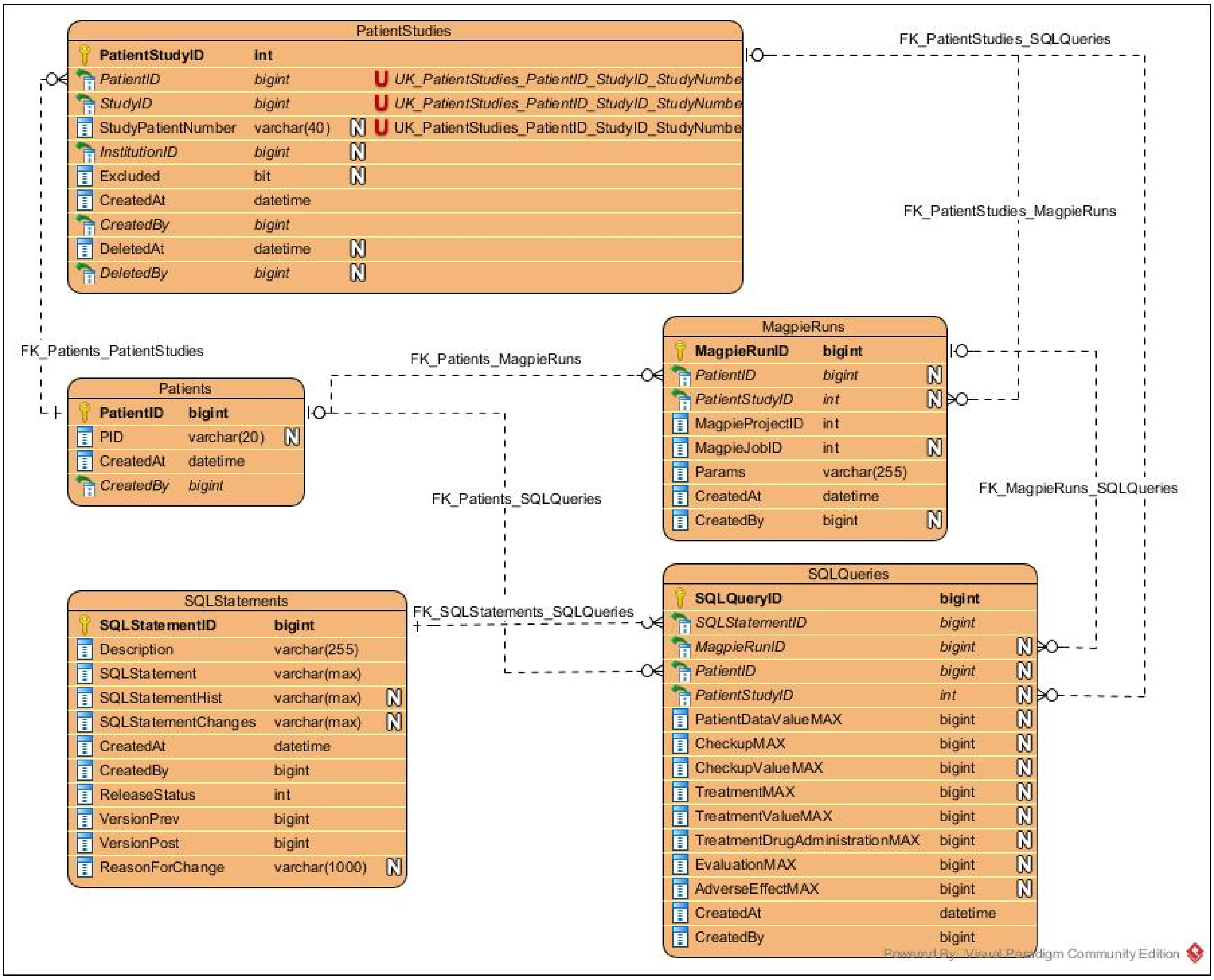

Fig. 9: ERD for management of simulation data

3.4 Access management

Fig. 10 depicts the ERD of the role-based access management system. User groups (e.g. physician, documentarist, administrator) and permission objects (e.g. Patients, Checkups, Treatments, Drugs, …) are predefined in tables *UserGroups* and *PermissionObjects*. The table *PermissionSets* contains the relationship between user groups and permissions (read, create, update, delete) regarding to permission objects.

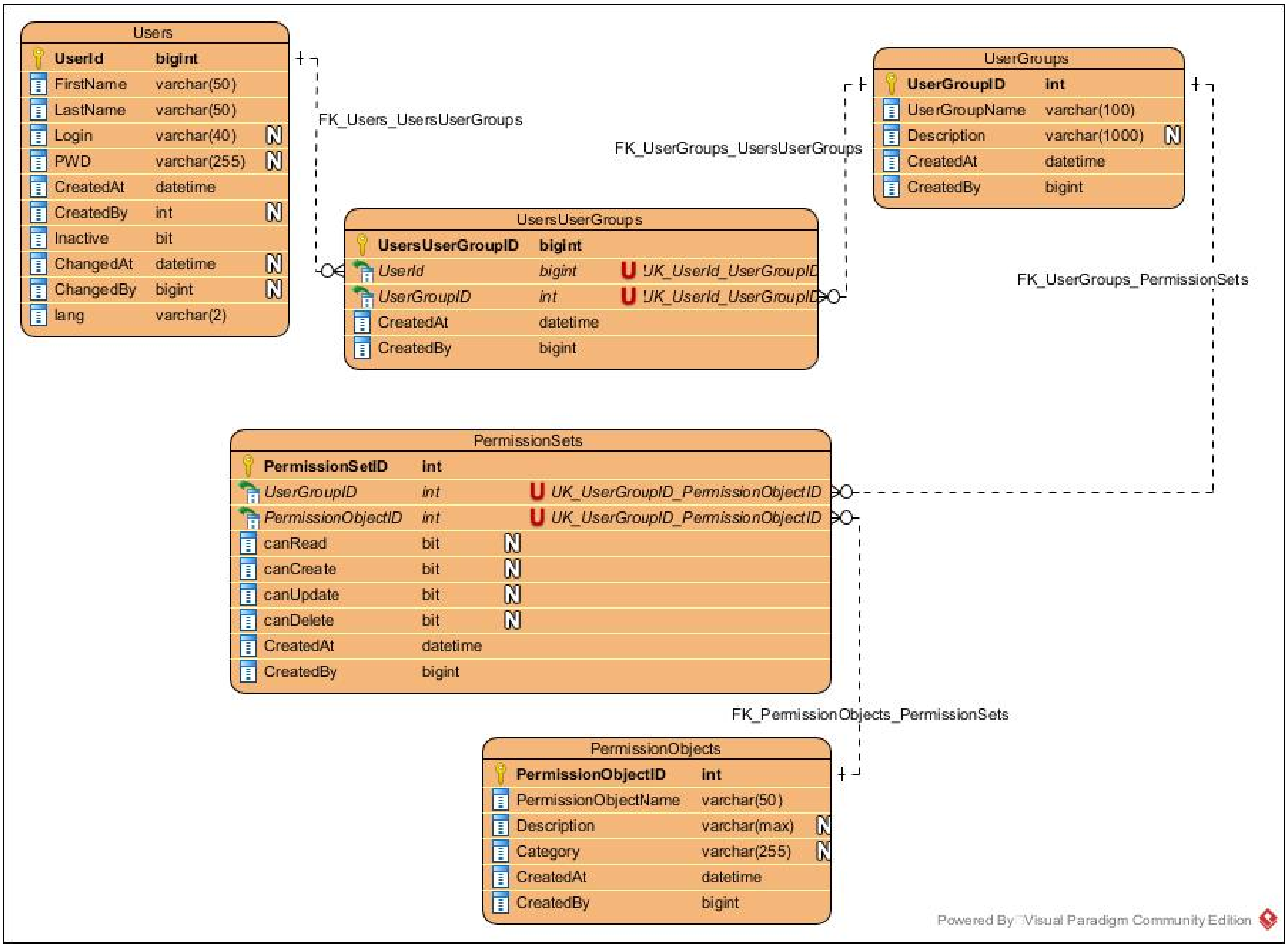

Fig. 10: ERD of the access control system

4 Traceability of data inserts, updates and deletes

The pseudonymized payload database is an insert-only database. If users execute the frontend's insert, update, or delete actions, a new record set with "parent-child-information" for traceability will be inserted. Therefore, every database table holding patient data is complemented with the following additional columns.

Table 1: columns with parent-child-information for traceability

Column	Content
ReleaseStatus	- encodes the release status of the record set<br>0: not released<br>1: released<br>2: deleted
VersionPrev	- signifies the previous record set by the primary key of the table<br>0: no previous dataset is available
VersionPost	- signifies the updated record set by the primary key of the table<br>0: no following dataset is available
